# Improving PMMA resin using graphene oxide for a definitive prosthodontic rehabilitation - A clinical report

**DOI:** 10.4317/jced.55883

**Published:** 2019-07-01

**Authors:** Luís Azevedo, José L. Antonaya-Martin, Pedro Molinero-Mourelle, Jaime del Río-Highsmith

**Affiliations:** 1 DDS. Postgraduate student, Department of Conservative Dentistry and Orofacial Prosthodontics. School of Dentistry. Complutense University of Madrid, Madrid, Spain; 2 DDS, PhD. Clinical Assistant Professor, Department of Conservative Dentistry and Orofacial Prosthodontics. School of Dentistry. Complutense University of Madrid, Madrid, Spain; 3 DDS, MDS. PhD student, Department of Conservative Dentistry and Orofacial Prosthodontics. School of Dentistry. Complutense University of Madrid, Madrid, Spain; 4 MD, DDS, PhD. Professor, Department of Conservative Dentistry and Orofacial Prosthodontics. School of Dentistry. Complutense University of Madrid, Madrid, Spain

## Abstract

The use of acrylic resins as a definitive material has shown some advantages comparing to other definitive materials. However, their poor mechanical properties remain a major drawback. In this case report, graphene oxide (GO) was incorporated into polymethyl methacrylate (PMMA) resin for a definitive maxillary rehabilitation, combined with an intraoral digital impression and a three-dimensional facial scan. After 8 months of the placement of the definitive prosthesis, no mechanical, aesthetic, or biologic complications were reported, and the soft tissues showed excellent health and stability. The incorporation of GO in PMMA resins seems to be a suitable option for prosthetic rehabilitation. However, further studies are needed to ensure rigorous scientific support of these techniques and materials.

** Key words:**Computer-aided design, computer-aided manufacturing, dental impression technique, dental prosthesis; graphene oxide.

## Introduction

Acrylic resins, mainly included polymethyl methacrylate (PMMA), have been used for a long time ago as a provisional material in prosthodontics. They present good properties, such as low modulus of elasticity (1), less wear of the antagonist (2), good aesthetic results (3), easier repair (4), economic cost and a relatively fast manufacturing process. However, their main disadvantages are their poor resistance to wear and tear (5), and volume shrinkage after the polymerization (6). Moreover, their lack of strength under fatigue failure (7), and the microbial adhesion onto PMMA (8) are a major drawback for their long-term use.

Graphene is a single sheet of one-atom thickness arranged in a honeycomb-like lattice (9). Each carbon atom is covalently bonded to three other carbon atoms with sp2 hybridization. While the interlayers are re-arranged through weak Van der Waal forces. These forces are responsible for the softness of this material (9). Graphene exists in different forms such as graphene sheets, graphene oxide (GO) and reduced graphene oxide (rGO).

GO’s properties, such as its biocompatibility and biodegradability (10), strength (Young’s modulus of Y ~ 1.0 TPa) (11), antimicrobial-adhesion characteristics (12), flexibility (11), and transparency make it a material with potential in prosthodontics.

Although there are several studies on the application of GO in dental/medical fields, mainly for bone tissue engineering and restorative dentistry (bone cements, scaffolds, biocomposites), to our knowledge there is only one dentistry study on GO incorporated into PMMA resins (12).

This clinical report shows the potential of GO in improving the mechanical properties of PMMA resin to be used as a definitive material for prostheses. It also describes the use of 3-dimensional (3D) facial and intraoral scanners to register the 3D information for a maxillary fixed tooth-supported rehabilitation.

## Case Report

A 75-year-old woman visited the Dental Clinic of the Faculty of Dentistry of the Complutense University of Madrid and requested restorative treatment for her compromised functional and aesthetic situation. Her medical history included a lung cancer ten years earlier.

**Figure 1:**
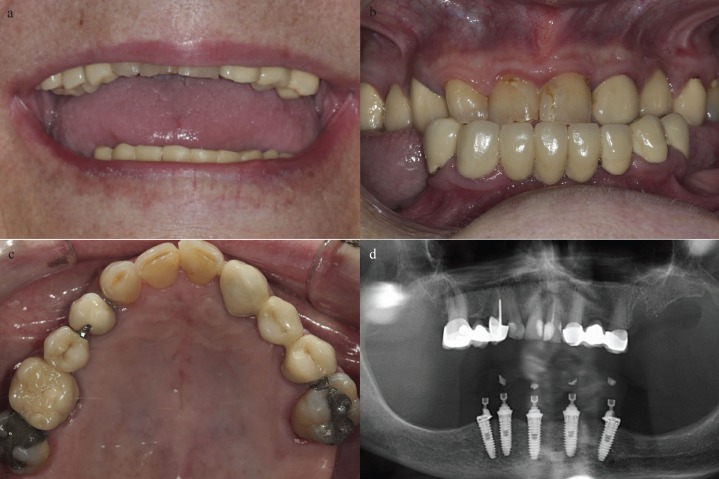
Initial clinical situation. A) Extraoral view of smile. B) Frontal view of maximum intercuspation. C) Occlusal view of the maxila. D) Panoramic radiograph.

The diagnostic criteria included clinical examination, intraoral pictures, radiographic examination and diagnostic cast assessment (Fig. 1).

The intraoral examination revealed two 3-unit maxillary fixed partial dentures (FPDs) supported by the first premolar and the first molar in the first quadrant and by the canine and the second premolar in the second quadrant. These FPDs had been placed due to the agenesis of the upper lateral incisors. The examination also showed extensive amalgam restorations in the right second molar and in the left first molar, which were extensively compromised due to an advanced process of dental root caries and furcation involvement.

The clinicians decided to extract the compromised molars and restore the other teeth with a fixed dental prosthesis, to increase the vertical dimension and replace missing dental pieces. For occlusal reasons and to avoid distal extensions in the lower prosthesis, no implants were placed. All preparations were made using a biologically oriented preparation technique (BOPT) of vertical preparation without finish lines.

After basic periodontal treatment (scaling and root planning), the maxillary right second molar and left first molar were extracted. Recommendations on oral hygiene were given. The sutures were removed 7 days after surgery.

One month later, after a healing period, a preliminary impression with an intraoral scanner (Trios, 3shape) was made for the design (Dental system, 3shape) and fabrication of the first provisional prosthesis in PMMA. The preliminary cast was 3D printed in a resin material (Formlabs Inc).

**Figure 2:**
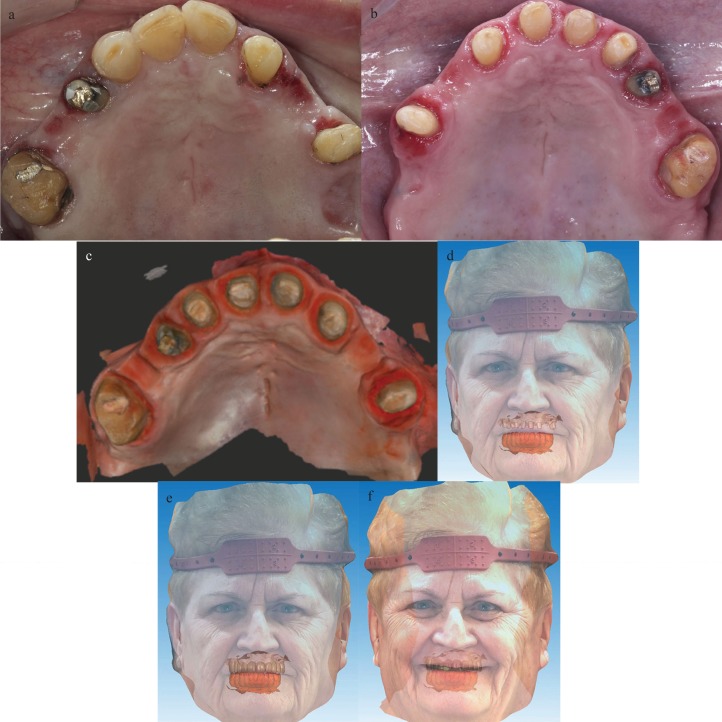
A) Occlusal view of the maxila after removing metal-ceramic restorations. B) Occlusal view of the maxila after vertical preparation. C) Digital impression of the prepared teeth after a healing period. D) Virtual image of the facial scan aligned with the digital intraoral impression of the prepared teeth in maximum intercuspation. E) and F) Virtual image of the facial scan aligned with the digital intraoral impression of the provisional prosthesis in maximum intercuspation and in smile position.

Subsequently, the existing metal-ceramic restorations were removed (Fig. 2A) and double probing was performed to measure the depth of the gingival sulcus and the location of the bone, so as to determine the limit of tooth preparation. All teeth were prepared with vertical preparation technique to eliminate the existing finish line, as described Agustín-Panadero *et al.* (13) (Fig. 2B). The splinted PMMA-based provisional prosthesis was cemented (Temp Bond Clear, Kerr) on the prepared teeth and the intrasulcular zone of the restoration margin was relined with composite to support the gingival margin circumferentially and to create the new emergence profile. The reinsertion and thickening of gingival tissue depended on the healing process (13).

The provisional prosthesis was maintained for 3 months, during which the prosthesis’ emergence profile was modified to achieve gingival adaptation.

After this period, the definitive impression was taken using another intraoral scanner (Medit i500, Medit) (Fig. 2C). A 3D facial scan was also obtained (AFT System One, AFT Dental System) to align and merge the facial scan with the digital cast for aesthetic evaluation (Fig. 2D,E,F).

**Figure 3:**
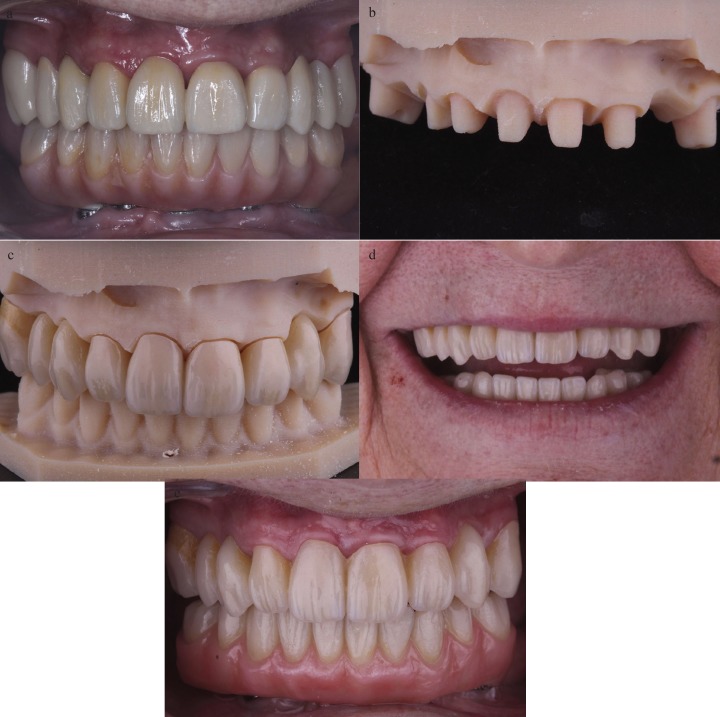
A) Wax try-in denture. B) 3D-printed working cast. C) Definitive fixed dental prosthesis made from PMMA reinforced with graphene. D) Post-treatment extraoral view of smile. E) Frontal view of the definitive maxillary fixed dental prosthesis made from PMMA reinforced with graphene.

With the information provided by the facial and intraoral scans a new wax try-in denture was designed, and 3D printed in a resin material (Formlabs Inc), to evaluate the aesthetic parameters, tooth positions and vertical dimension (Fig. 3A). A new cast was 3D printed in a resin material (Formlabs Inc) (Fig. 3B).

The definitive fixed dental prosthesis was made based on the biologic and functional parameters of the provisional restorations. A prosthesis made from PMMA resin reinforced with graphene oxide was designed (Exocad software, exocad GmbH) and milled (Fig. 3C). Passive fit, interproximal contacts and occlusion were checked in the patient´s mouth.

After all the parameters were confirmed, the prosthesis was cemented with a dualpolymerizing resin cement (Variolink Esthetic DC, Ivoclar Vivadent) that was then light-polymerized (Fig. 3D,E). The patient was given oral hygiene instructions and information on how to take care of the new prosthesis.

Follow-up evaluations were made 1 week and 1, 3 and 8 months after the placement of the definitive prosthesis. No mechanical, aesthetic, or biologic complications were reported, and the soft tissues showed excellent health and stability.

Patient informed consent was obtained for the publi¬cation of this article

## Discussion

The present clinical report used GO as a novel additive in PMMA resins to overcome the resin’s mechanical properties. According to several authors, the incorporation of carbon nanotubes, such as GO, in acrylic resins may enhance the resin’s mechanical properties (14) and decrease the degree of contraction during polymerization (15). Furthermore, Lee et al. (12) reported that PMMA exhibited better antimicrobial-adhesion effects after incorporating GO than those exhibited by pure PMMA via increased hydrophilicity.

Taking into account the reduced follow-up time of this clinical report, it seems that the incorporation of GO in PMMA resins seems to be an interesting strategy to improve their mechanical properties and provide a definitive, reliable material option. However, further studies are necessary to ensure rigorous scientific support and to determine the benefits of these new technologies and materials.
